# Perspective on Adenoviruses: Epidemiology, Pathogenicity, and Gene Therapy

**DOI:** 10.3390/biomedicines7030061

**Published:** 2019-08-19

**Authors:** Brennetta J. Crenshaw, Leandra B. Jones, Courtnee’ R. Bell, Sanjay Kumar, Qiana L. Matthews

**Affiliations:** 1 Microbiology Program, Department of Biological Sciences, College of Science, Technology, Engineering and Mathematics, Alabama State University, Montgomery, AL 36104, USA; 2 Departments of Pediatrics and Cell, Developmental and Integrative Biology, Division of Neonatology, University of Alabama at Birmingham, Birmingham, AL 35233, USA; 3 Department of Biological Sciences, College of Science, Technology, Engineering and Mathematics, Alabama State University, Montgomery, AL 36104, USA

**Keywords:** adenovirus, serotype, Mastadenovirus, epidemiology, gene therapy, exosomes

## Abstract

Human adenoviruses are large (150 MDa) doubled-stranded DNA viruses that cause respiratory infections. These viruses are particularly pathogenic in healthy and immune-compromised individuals, and currently, no adenovirus vaccine is available for the general public. The purpose of this review is to describe (i) the epidemiology and pathogenicity of human adenoviruses, (ii) the biological role of adenovirus vectors in gene therapy applications, and (iii) the potential role of exosomes in adenoviral infections.

## 1. Introduction

**Figure 1:**
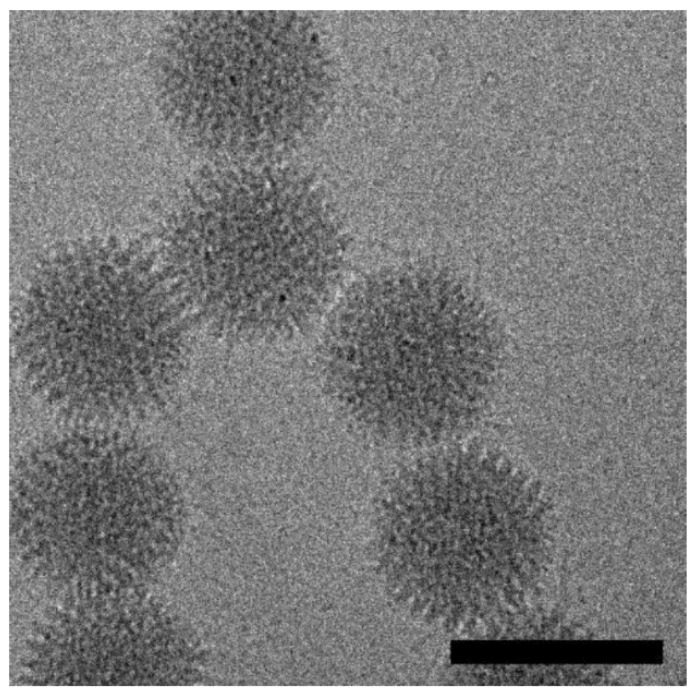
A digital cryo-electron micrograph of the Adenovirus serotype 5 (Ad-5)/HVR5-33RGD vector. Adenoviruses are non-enveloped, double-stranded DNA viruses that cause cold-like infections of the upper respiratory tract. These viruses have an icosahedral-shaped capsid that ranges from 90–100 nm in diameter and a ~36-kb genome. This image was collected on a Tecnai-12 microscope using a Gatan UltraScan 1000 (2k × 2k) CCD camera. The scale bar represents 1,000 Å. Image provided courtesy of Dr. Phoebe Stewart, Case Western Reserve University, Cleveland, OH, USA.

Human adenoviruses (HAdVs), which are members of the family *Adenoviridae* and genus *Mastadenovirus*, are non-enveloped, icosahedral, double-stranded DNA viruses (Figure 1) [1,2,3] that were first isolated from human adenoid tissue cultures in 1953 by Wallace Rowe and colleagues while studying the growth of polioviruses in adenoidal tissues [4,5,6]. HAdV species are classified into seven groups (HAdV-A to HAdV-F; Table 1) [6,7], and to date, 67 HAdV serotypes have been reported [6,7]. Of these, only some cause severe infections, leading to meningitis, conjunctivitis, gastroenteritis, and/or acute hemorrhagic cystitis [6,8,9,10,11]. Most of these infections occur in children [12,13], the elderly [14,15], and people with a severely compromised immune system [7,16,17]. The association of HAdV serotypes with a specific disease, however, has not been fully elucidated, as the clinical manifestations are sometimes linked to the site of viral inoculation [8,18]. Furthermore, due to the infectious nature of these viruses and the use of exosomes as a cellular mechanism of entry, HAdV is suited to provide efficacious gene therapy and facilitate drug delivery for diseases, including cancer. The purpose of this review is to describe (i) the epidemiology and pathogenicity of HAdVs, (ii) the biological role of adenovirus (Ad) vectors in gene therapy applications, and (iii) the potential role of exosomes in adenoviral infections.

## 2. Epidemiology

HAdV infections are readily spread in human populations [10]. Outbreaks often occur in crowded populations, such as nosocomial facilities (e.g., hospitals and nursing homes) [15,19,20], military bases [21,22], and schools [23,24]. HAdVs may cause outbreaks of diarrheal and gastroenteritis illness [25], pharyngoconjunctival fever [26], febrile respiratory illness [27], and/or keratoconjunctivitis [28]. A person infected with HAdV is extremely contagious during the incubation period, which typically ranges from 4–8 days, but can last up to 24 days, depending on the HAdV serotype [7].

HAdVs are grouped into seven groups (A–G) and 67 serotypes (1-67) in the genus *Mastadenovirus* (Table 1) based on their physical, chemical, and biological properties [6,7]. HAdV serotypes 3, 4, 7, 8, 14, and 55, which are commonly linked to outbreaks, are more virulent and likely to spread [6,29,30,31,32,33,34]. Different HAdV serotypes exhibit different tissue tropisms and clinical manifestations of infection [29,35]. Additionally, the predominant serotypes detected in association with disease differ among different countries or regions and change over time [29]. HAdV strains can actually cross continents, replacing old strains with new strains and changing the dominance of a serotype in a geographical region. Given the abundance of outbreaks that have occurred globally, we will focus on a description of the outbreaks that have occurred in the U.S.

HAdV outbreaks do not occur frequently in the U.S.; however, when outbreaks do occur, they spread rapidly among the human population. According to the Center for Disease Control and Prevention (CDC) National Adenovirus Type Reporting System, ~2400 cases of HAdV were reported in the United States (U.S.) between 2006 and 2016, the most recent period for which data are available. However, since 2016, a number of HAdV-associated outbreaks have recently occurred in the U.S. between September and November 2018. In September 2018, HAdV-7 outbreak occurred at the Wanaque Center for Nursing and Rehabilitation in Wanaque, New Jersey [36,37,38], infecting ~35 people, including 23 children. Of these 23 children, 11 reportedly died due to the illness [36,37]. Another HAdV-associated (HAdV-3) outbreak occurred in New Jersey at the Voorhees pediatric facility (Camden County) in November 2018. Twelve cases were reported, but no deaths occurred as a result of the outbreak. 

Similar to the outbreak at the Wanaque Center, HAdV-7 was reported on November 2018 at the University of Maryland (College Park, MD, USA) [39,40]. By mid-December, 35 cases were reported [39,40]. According to sources from *The Washington Post*, one student who was taking medication for Crohn’s disease died from the illness [39,40]. Currently, the CDC provides technical assistance for testing and typing specimens and consultation on infection control for HAdV outbreaks in the U.S. [37].

## 3. Pathogenesis

HAdVs are very stable in the environment. Some HAdVs spread via local outbreaks in common areas, such as summer camps [50], playgrounds, dormitories [40], and schools [23]. Transmission occurs from an infected person to other individuals via respiratory routes, fecal-oral contamination, and/or direct contact [51]. Respiratory transmission via a cough or a sneeze is the most common mode of transmission. Fecal-oral transmission occurs through contaminated food or water, and transmission via water can occur in public swimming pools due to ineffective chlorine treatment [51]. HAdV infection can also occur through an individual’s lack of proper hygiene, such as improper handwashing. HAdVs can infect and replicate in epithelial cells of the gastrointestinal (GI) tract, respiratory tract, eyes, and urinary bladder [1]. HAdVs cause lytic infection in epithelial cells and/or latent infection in lymphoid cells [6,52]. Different serotypes have different tropisms related to their route of infection and receptor usage.

The HAdV genome is divided into the early (E), intermediate (I), and late (L) regions [7,53]. The E region of the genome consists of the transcription units E1 to E4, which are required for viral replication and modulation of host immune response. The I region of the genome contains the transcription units IX, which influences hexon protein interactions, and IVa2, which is involved in viral DNA packaging and virus assembly and is a transcriptional activator [53,54]. The L region of the genome comprises the L1-L5 transcription units, which are involved in the production of mature virions [7]. Additionally, the HAdV genome displays inverted terminal repeat regions at the 3′ and 5′ ends, encompassing conserved sequence motifs and serving as origins of viral replication [7]. Depending on the HAdV serotype, the genomes may display noncoding virus-associated RNA genes that are involved in translational regulation and potentially act as miRNAs [7,55,56].

HAdV-2 and 5 attach to the surface of cells with their fiber proteins via the coxsackievirus and adenovirus receptor (CAR), which is a 46-kDa transmembrane protein [57,58,59]. CAR is involved in the formation of tight junctions and adheres junctions between epithelial cells [60,61] and interacts with the fiber knobs from all HAdV, except those from group B [62]. However, this high-affinity receptor interaction is unable to promote virus entry into cells. Instead, a secondary interaction between the virus penton base protein and αvβ3 or αvβ5 integrins facilitate virus entry [63]. HAdV particles enter cells via ~120-nM clathrin-coated pits and vesicles [64], although internalization also requires the participation of cell signaling molecules, including phosphatidylinositol 3-OH kinase, a lipid kinase that regulates a number of important host cell functions [65]. A crucial HAdV entry step involves post-internalization disruption of the early endosome, allowing the escape of the virion from the cytoplasm prior to destruction by lysosomal proteases [66]. Once inside the cell, the virus is transported to the nucleus and docks at the nuclear pore, where capsid uncoating reveals the viral DNA.

For most other HAdV types, the attachment receptor is either CAR or CD46, which is a membrane cofactor protein [67]. The precise mechanism for HAdV binding to blood and epithelial cells was unknown [68], but HAdV-3, -7, -11, -16, -21, -26, -35, -37, -49, and -50 were recently reported to bind to membrane cofactor CD46 [69]. CD46 is expressed on all nucleated cells in humans and functions to shield autologous cells from complement attack [70]. Binding of CD46 to HAdV is mediated by fiber knobs, which recognize CD46 with different affinities. Furthermore, other identified HAdV attachment receptors include sialic acid-containing oligosaccharides, GD1a glycan, and desmoglein-2 [57,71,72]. Low-affinity, high-avidity binding allows viruses to use multiple receptors, depending on the receptor availability and expands virus tropism. 

HAdVs cause either lytic infection in epithelial cells or latent infection in lymphoid cells [6,52]. The lytic infection is referred to as the viral reproduction cycle. The lytic infection occurs when the Ad enters and replicates inside of the host (human epithelial cells). The virus can inhibit the macromolecular synthesis and transport mRNA to the cytoplasm of the cell, facilitating cellular death and cell lysis. After the virus actively replicates inside of the host cell, it causes cellular death and cell lysis. In addition, virions are produced, resulting in a host inflammatory response [6,73]. 

**Figure 2:**
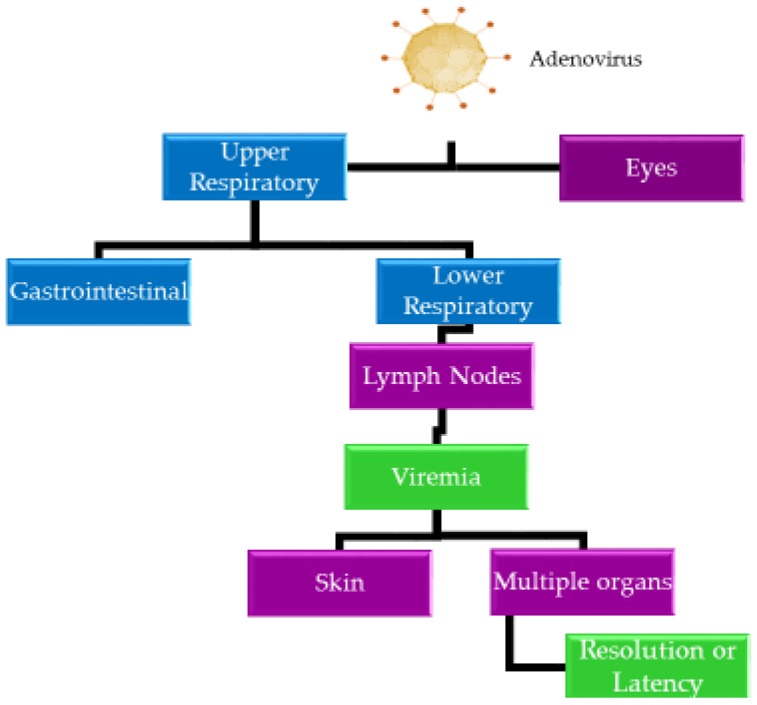
Mechanisms of HAdV spread within the body. HAdV may be transmitted directly or indirectly. These viruses can adversely impact body systems and cause organ dysfunction. HAdV can evade the immune response and produce persistent or latent infections. The organs are represented in purple, while the body systems are represented in blue. The biological status of the infected host is indicated in green.

Following the lytic infection, HAdVs can persist in susceptible cells in a latent state for years [7]. During latent infection, HAdVs generally remain in lymphoid organs, such as adenoids, tonsils, or Peyer’s patches [52,74]. These latent virus particles can eventually re-activate, re-infect, and replicate in epithelial cells, causing disease symptoms again. Figure 2 illustrates the spread of HAdVs within the body.

## 4. Diagnosis, Treatment, and Prevention of Adenoviral Infections

The most common method used to diagnose a HAdV infection starts with a clinical evaluation of an individual’s symptoms. Occasionally, doctors will use chest X-rays, nasal swabs, and/or blood or stool cultures to confirm an HAdV diagnosis. Laboratory diagnosis is mainly performed to detect and prevent large outbreaks. Some of the laboratory diagnostic techniques include antigen detection, molecular detection, viral isolation, and serology tests [75,76,77,78,79]. Like other viruses, HAdV contains many proteins, including hexon protein, fiber protein, and penton protein, on its surface. These proteins can serve as antigens for the host immune response, thereby inducing a defense mechanism to help the host fight off HAdV infection. Antigen detection methods, such as the enzyme-linked immunosorbent assay (ELISA) and latex agglutination test (LAT), are used to identify HAdV-associated proteins in patient samples. ELISA is a rapid, quantitative, sensitive, and specific diagnostic technique that is used to detect antigens from cells, bacteria, and viruses [79,80,81]. HAdVs can be detected in biological samples using commercially available ELISA kits, including the Adenovirus ELISA kit (Abnova), Adenovirus ELISA kit (antibodies-online.com), Adenovirus IgM ELISA kit (GenWay), Anti-Adenovirus 3 Antibody (IgG) ELISA kit (Lifespan Biosciences, Seattle, WA, USA), and the Adenovirus Antigen ELISA kit (Eagle Biosciences, Amherst, NH, USA). The Adenovirus Antigen ELISA Assay Kit is a microplate-based kit that is used to qualitatively detect HAdV antigen in feces. This assay is often used to diagnose active HAdV infection in acute or chronic gastroenteritis. For example, in 2016, Mayindou et al. utilized ELISA kits to investigate the prevalence of severe diarrhea in Congolese children that were hospitalized with severe acute gastroenteritis as a result of Ad serotypes 40 and 41 and Rotavirus a [79]. Additionally, LAT is used to detect HAdV infections in saliva, urine, blood, or cerebrospinal fluid samples [82,83,84] and detects HAdV via binding with latex beads coated with a specific antigen or antibody [85]. The first reported use of LAT to detect HAdV was performed in 1987 by Grandien et al. to detect HAdV serotypes 40 and 41 in diarrheal disease in children [82]. Moreover, in 1993, Lengyel et al. demonstrated through the LAT that latex-coated particles with different monoclonal antibodies of genus-specific reactivity can be used to rapidly diagnose HAdV infections [83].

In addition to these techniques, molecular detection of HAdV infection is often performed using the polymerase chain reaction (PCR) assay [7,86,87,88], which amplifies small segments of viral DNA, enabling laboratory technicians and doctors to detect the presence of HAdV in blood, stool, and/ or mucous samples [89,90]. In 2017, Bennett and Gunson developed a single multiplex assay to detect viral gastroenteritis from patients’ stool samples [87].

Viral isolation from cell culture is another standard method used to detect HAdVs in respiratory and conjunctival specimens [91]. HAdVs may be isolated from most bodily fluids and secretions, including eye swabs, throat swabs, urine, and feces; however, the most reliable source for isolation is feces. Although this method is sensitive, the time interval between inoculation and manifestation of cytopathic effect (CPE) is often variable, based on the concentration of an infectious virus or the serotype of HAdV in the clinical specimen. In specimens with small quantities of virus, CPE may be delayed for as many as 28 days. Furthermore, in some cases, viral isolates are not able to be cultured, suggesting that this method is not always conclusive.

Serology tests are performed to assess the levels of antibodies (i.e., IgG enzyme-linked immunosorbent assay (EIA) and IgA EIA) generated against active infection with HAdV. Serum and plasma from possible infected individuals are collected and tested for HAdV infection, using commercial kits, such as the Ad R-Gene kit (Biomerieux Diagnostic), ELITe MGB kit (ELITe Tech Group, MD, USA), and Film Array RP kit (BioFire Diagnostics) [7]. In addition to HAdVs, most of these kits are used to detect other viruses (e.g., influenza A and B viruses) [92,93]. Advantages of these kits include sensitivity and specificity, ease of use, simple interpretation, the requirement for minimal sample for testing, and rapid turnaround time; yet, these serological tests can be less sensitive than culture.

In general, HAdVs are resistant to low to intermediate levels of disinfectants, such as ethanol and chlorine, as well as to heat inactivation (≤60 °C) [94,95]. Therefore, HAdVs can remain on objects and surfaces (e.g., doorknobs, towels, and medical instruments) for ~3-8 weeks, posing long-term infection risk [94,95,96]. Therefore, appropriate control measures should be taken into consideration to minimize the transmission of HAdV infections and prevent outbreaks. These individual control measures include frequent handwashing, sanitizing surfaces, staying at home when ill, avoiding close contact with people who are sick, and covering nose and mouth when sneezing or coughing.

No specific treatment for HAdV infection has been developed [96]. As most HAdV infections are mild and do not require medical care, clinical care of HAdV infections focuses on alleviating patient symptoms. Commonly recommended treatments often include bronchodilator medication to open the airways, oral rehydration or increased fluid intake, and rest. In addition, antiviral drugs, such as ribavirin and cidofovir, have been used to treat severe HAdV infections in immunocompromised people [29,94].

According to the U.S. CDC, a vaccine for HAdV is not currently available to the general public in the U.S. A vaccine is only available for U.S. military personnel, ages 17–50, who may be at higher risk for acute respiratory disease related to infection with HAdV serotypes 4 and 7 [34,97]. From 1971 to 1999, a vaccine against these two serotypes was available to U.S. military recruits [98], but in 1999, the manufacturer stopped producing this vaccine [94,98,99]. In March 2011, the U.S. Food and Drug Administration approved a new live, oral vaccine against HAdV serotypes 4 and 7 [94,97,99], and this vaccine is recommended by the USA. Department of Defense for military recruits entering basic training in order to prevent acute respiratory disease [94]. For military recruits, the vaccine is highly recommended because of the close military living quarters and easy transmission.

## 5. Ad Vectors in Gene Therapy

Various Ad vector systems have been studied for use as gene therapy in clinical trials. Ad vectors have been studied widely and are well characterized as a model system for eukaryotic gene regulation, providing a solid foundation for human gene therapy vector development. Accordingly, applications of Ad vectors in gene delivery have greatly increased since their initial development during the late 1980s [100]. More than 2000 gene therapy clinical trials have been approved worldwide [100,101,102,103]. There are many advantages of utilizing Ad vectors as viral gene delivery systems: (i) these viruses are easy to manipulate and generate, (ii) they can be grown into stable high-titer stocks for repeated use, (iii) they infect non-dividing and dividing cells of different types, and (iv) they infect a broad host range with high infectivity. Due to the life cycle of Ad, the virus does not require integration into the host cell genome, and the foreign genes delivered by Ad vectors are expressed as an episome, imparting low genotoxicity in vivo. The first successful in vivo gene therapy using Ad vectors in humans was reported by Jaffe et al. in 1992 [104,105]. In these studies, Ad vector was used to deliver and express alpha-1 antitrypsin cDNA in the liver cells of a patient with low levels of this factor [104,105], confirming that Ad vectors could potentially be used for gene therapy for liver disorders in vivo. This proof-of-principle by Jaffe et al. led to the development of additional Ad vectors for use in gene therapy applications against diseases, such as cancer. Examples of Ad vectors used in gene delivery clinical trials are listed in Table 2. 

HAdV vectors are used for virotherapy and gene therapy for cancer [101,106,107,108]. The application of HAdVs for cancer therapy dates back to the 1950s when wild-type Ad was used to treat cervical cancer. Due to high infectivity, cytotoxicity, and immunogenicity, Ad vectors were pursued as anti-cancer therapeutics [101,109,110]. Replication-defective Ads have been used to deliver immune-related genes/epitopes directly to tumor cells to attract and induce a local anti-tumoral immune response [100,111,112,113,114,115,116,117,118], while replication-competent Ads have been used to replicate within cancer cells, achieving oncolysis via exploitation of the natural lytic life cycle of the virus within these cells [100,101,119,120,121]. Thus, either replication-defective or -competent Ads can be used to deliver and/or overexpress tumor-suppressor genes, antisense oncogenes, or cytotoxic/suicide genes in cancer cells to directly induce an intrinsic cytotoxic cascade, cause cell cycle arrest, or trigger apoptosis as an anti-tumor agent [100,101,122], and such delivery has been shown to be effective in inducing tumoricidal effects and anti-cancer immunity in different animal models [109,123,124,125]. For example in 2003, Gendicine, which is a recombinant Ad that expresses wild-type p53 from a Rous Sarcoma virus promoter, became the first licensed gene therapy product in China to treat cancer [100,101,122]. Based on the fact that p53 is one of the most widely studied tumor suppressors, this gene provided an ideal target for gene replacement therapy [109,126]. Following cellular stress conditions, p53 induces senescence, cell cycle arrest, DNA repair, autophagy, and/or apoptosis [127]. Thus, Gendicine has been approved to treat patients with head and neck cancers [127,128,129]. Similar to Gendicine, Advexin is another commonly used Ad-based anti-cancer drug [100,130,131]. Advexin is an E1-E3-deleted HAdV-5 vector that expresses p53 from a cytomegalovirus promoter in the E1 region [100]. Advexin has been used in numerous cancer treatments, including head and neck cancer, prostate cancer, colon cancer, and breast cancer [100,132,133,134].

In general, HAdV-5 vectors are the most commonly used vectors for cancer gene therapy [109]. In 2014, Azab et al. reported that Ad-5/3 cancer terminator virus suppressed tumor growth in a nude mouse xenograft model and in a spontaneously induced prostate cancer in Hi-myc transgenic mice [135]. Another HAdV-5 vector was reported in 2016 by Gu and colleagues. This group developed an Ad5H3 chimera using the antigen capsid-incorporation strategy, and this alternative vaccination approach induced an antigen-specific humoral immune response to escape HAdV-5 neutralization [136].

Although these viral vectors offer many advantages, several major limitations must be addressed in order to effectively utilize Ad vectors for successful gene therapy in human patients. These challenges include high levels of pre-existing immunity in patients, transient transgene expression, high immunogenicity, and induction of potent inflammatory responses [104,137,138]. In addition, some HAdVs have limited infectivity in some cancer cells. As a result, extensive efforts have been made to address these limitations. Researchers have developed alternative methods, such as altering the tropism of HAdVs, vector chimeras, cytotoxic/suicide gene therapy, and combination immunotherapy approaches, to induce a better anti-cancer effect [107,139,140,141,142].

## 6. Role of Extracellular Vesicles (EVs) in Ad Infection

Viruses often enter host cells through interactions of viral ligands with cellular receptors [145,146]. Receptor-mediated virus entry has been studied in-depth for quite some time; however, mechanisms regarding receptor-independent viral entry into cells have not been fully explained. According to the Trojan hypothesis, retroviruses exploit pre-existing pathways for intracellular trafficking for host cell entry [147,148]. These pathways involve the non-viral exosome biogenesis pathway for the formation of infectious particles and the pre-existing, non-viral pathway for exosome uptake as a receptor-independent, envelope-independent mode of infection [148,149,150,151]. Moreover, it has been proposed that the release of small, membrane-derived EVs, termed exosomes, may offer a mechanism by which viruses, including HIV and HAdVs, enter cells via receptor-independent entry [146,148,152]. 

Exosomes are nano-sized, membrane-bound vesicles that range from 40–150 nm in diameter. Exosomes are released into the extracellular microenvironment by all types of eukaryotic cells, including epithelial cells, glial cells, and neurons, as well as a few prokaryotic cells, including bacteria [152,153,154]. These discharged vesicles have been observed in blood, urine, semen, saliva, cerebrospinal fluid, and breast milk and play a central role in intracellular communication via their involvement in key biological processes [152]. The composition of these small vesicles reflects the composition of the subcellular origin and the physiology of the parent cells [154,155]. Exosomes have been found to be involved in several pathophysiological processes, such as neurodegenerative disorders, infectious disease, cardiovascular disease, and cancers [154,156,157,158]. Recent studies have explored potential roles for exosomes in the pathogenesis of portal hypertension, fibrosis, and liver inflammation [159,160,161]. 

Biogenesis of exosomes has not yet been fully elucidated; however, current literature suggests that exosomes are formed when early endosomes mature into late endosomes [152,162]. The late endosomes then form into multivesicular bodies, which fuse with the plasma membrane and release their contents into the extracellular environment [152,162]. Exosomes are secreted via the constitutive or inducible release pathway [163]. In the constitutive release pathway, proteins (e.g., Rab guanosine triphosphatases) are sorted into vesicles in the Golgi, transported to the cell surface, and fused with the plasma membrane through exocytosis [163]. In the inducible release pathway, stimuli, such as hypoxia, DNA damage, and heat shock, are regulated [163]. Exosomes act as carriers to transport DNA, various types of RNAs, lipids, and proteins [164,165,166,167,168]. These vesicles are enriched with tetraspanins (e.g., CD9, CD63, and CD81) and endosome-associated proteins (e.g., annexin and Rabs) [161,169,170]. A more detailed description of the molecular constituents found within exosomes is reviewed in Crenshaw et al. [163].

**Figure 3:**
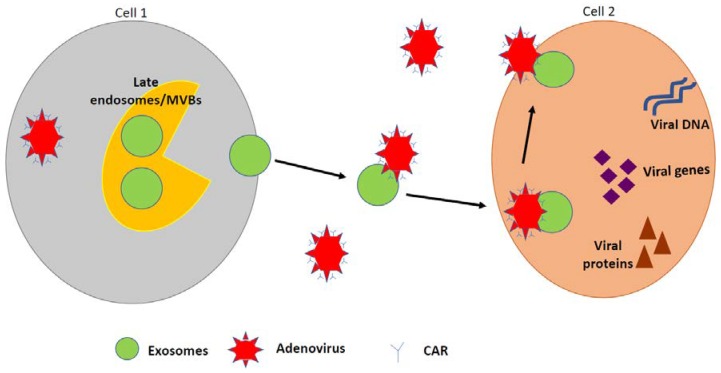
Exosomes mediate HAdV attachment and entry. The coxsackie and adenovirus receptor (CAR) mediates the entry of HAdv into human cells. Exosomes act as carriers for viruses, and this inclusion is important for viral survival, spread, and infection in the host organism. The HAdV binds to exosomes, enters cells via phosphatidylserine/TIM-4 interactions or other binding proteins. In addition, the virus injects their DNA into the host cell, which produces copies of its genes and proteins, resulting in transmission. Multivesicular bodies (MVBs).

Recent findings suggest that exosomes can carry viral genomes and act as cargo for viruses. This is important for viral survival, spread, and infection in the host organism [146,152,156,157,171,172]. Enveloped and non-enveloped viruses have evolved to enter host cells and hijack host cellular activities [146,152,173,174,175]. This unique way of entry may be mediated by exosomes, which provide a mechanism for the virus to evade the host immune system [174,175,176]. Although, the Trojan hypothesis and viral protein trafficking within exosomes have been widely accepted for RNA viruses and exosomes, much less is known with respect to DNA viruses and exosomes. We have shown that HAdV-5 exploits exosomes for receptor-independent cellular entry [146]. Specifically, the T-cell immunoglobulin mucin (TIM) protein-rich exosomes aid in exosome-mediated viral genome entry. This mechanism occurs with DNA viruses, such as HAdV [146,157]. Previous studies demonstrated that exosomes significantly enhanced HAdV-5 entry in CAR-deficient cells, in which HAdV-5 had only very limited entry. These exosomes were found to contain TIM-4, which binds phosphatidylserine. Treatment with TIM-4 antibody significantly blocked exosome-mediated HAdV-5 entry [146]. In addition, we further speculate that exosomes are released in the serum of HAdV-infected cells and that these HAdV-derived exosomes release DNA, miRNA, RNA, and viral proteins that aid in intracellular communication between neighboring cells. A proposed schematic of HAdV utilization of exosomes to enter the host cell is shown in Figure 3.

## 7. Role of EVs and Ad in Therapeutic Applications

In contrast to their role in viral pathogenesis, exosomes can contribute to the diagnosis of infectious disease and cancer and mediate drug delivery [177,178,179]. Previously, it was demonstrated that HAdV-5 binds to neural stem cell-derived exosomes and is delivered to the brains of mice [152]. This study presented important information for the use of HAdV-5 as a potential gene therapy tool, demonstrating that exosomes derived from neuronal cells can mediate Ad transduction in vivo. The demonstration of exosome-mediated viral delivery is important for transduction of cells that are Ad-resistant and/or in certain in vivo situations. Zhu et al. sought to inhibit porcine reproductive and respiratory syndrome (PRRS) infection by blocking PRRS receptor binding [180]. These investigators demonstrated a significant additive anti-PRRS effect of two recombinant Ad vectors that were incubated and co-administered with artificial miRNA-containing exosomes and further proved that exosomes were efficient delivery systems for small RNA in pigs [180]. 

Other groups have also reported potential therapeutic uses for EVs and Ad-based vectors. In 2016, Ran et al. demonstrated that tumor cell-derived microparticles, a specific class of EVs, could serve as a carrier for oncolytic Ads, leading to highly efficient cytolysis of tumor cells for in vivo treatment efficacy. In these studies, the benefits of harnessing the anti-tumor effects of oncolytic Ads and tumor microparticles included the avoidance of pre-existing antibody immunity of the host, receptor-independent virus entry into tumor cells, promotion of nuclear entry of oncolytic Ads to stem-like tumor-repopulating cells [181]. Similarly, Garalo et al. demonstrated that human lung cancer cell-derived EVs could be used for systemic delivery of oncolytic virus (OV) and the chemotherapeutic agent paclitaxel, resulting in enhanced anti-tumor effects in a nude mouse model [182]. More recently, in a separate study, Garofalo and colleagues used in vivo and ex vivo imaging to validate the cancer tropism attained when OVs are encapsulated inside EVs following intravenous administration but not intraperitoneal administration. This study further showed that the encapsulation of the virus did not disrupt virus function [183].

The use of EVs and Ads or OVs for therapeutic applications for cancer treatments has several advantages, including avoidance of the host immune response and expanded tropism of virus delivery. Although EVs have been shown to be safe in humans and have been approved for clinical trials, one of the biggest drawn backs to EV use is the lack of universal protocols for EV production for use as a drug delivery tool. Thus, a standardized protocol is urgently needed to expand the use of this system for therapeutic applications [184]. Additionally, the low production yield of EVs together with a short half-life following intravenous administration is a tremendous challenge that needs to be overcome before clinical applications can move forward [184,185,186,187]. Finally, additional studies to investigate possible immunogenic properties or toxic effects of EVs are warranted [188].

## 8. Conclusions

HAdVs are complex and evolving organisms. While much is already known about the epidemiology, structure/function, and pathogenicity of these viruses, a better understanding is needed to prevent the infectivity and spread of HAdVs. The superior plasticity and infectious nature of Ad vectors have positioned them as the most used viral vector for gene therapy and clinical biomedical research. The relationship between HAdV infection and EVs may provide a pivotal feature for the development of innovative cell-based therapies for diseases, including cancer. Furthermore, new information about the relationship between HAdVs and exosomes may also help to define new viral entry pathways that will lead to modalities to reduce infection.

## Figures and Tables

**Table 1:** Human adenoviruses (HAdV)-associated diseases or infections. HAdVs are members of the family *Adenoviridae* and genera *Mastadenovirus* and are associated with an array of diseases. HAdV is classified into seven groups (A–F). There are more than 100 serotypes, and approximately 67 serotypes (1–67) are known to be pathogenic in humans.

Group	Serotype	Associated Disease or Infections	References
A	12, 18, 31, 61	gastrointestinal, respiratory, urinary, cryptic enteric infection, linked to obesity, meningoencephalitis	[7,41,42,43,44]
B	3, 7, 11, 14, 16, 21, 34, 35, 50, 55, 66	conjunctivitis, gastrointestinal, respiratory, urinary, pneumonia, meningoencephalitis, cystitis	[7,41,42,44,45,46,47]
C	1, 2, 5, 6, 57	respiratory, gastrointestinal, obesity, pneumonia, hepatitis	[7,41,42,45]
D	8–10, 13, 15, 17, 19, 20, 22–30, 32, 33, 36–39, 42–49, 51, 53, 54, 56, 58-60, 63-67	conjunctivitis, gastrointestinal, linked to obesity, meningoencephalitis	[7,42,43,45,48]
E	4	conjunctivitis, respiratory, pneumonia	[7,41,47]
F	40, 41	gastrointestinal, infantile diarrhea	[7,42,49]
G	52	gastrointestinal	[7,42]

**Table 2:** Ad vectors currently used in gene therapy clinical trials in the U.S. [143,144].

Adenoviral Vector	Phase	Transgene	Condition	Administration Route	Clinical Trial Identifier
Ad-CCL21-DC	I	Serotype 5/C-C motif chemokine ligand 21 (CCL21) cDNA	Dendritic cells Advanced non-small cell lung cancer	Intratumoral	US-1720 NCT03546361
ETBX-071	I	PSA/MUC1/brachyury	Prostatic neoplasms Prostate cancer	Subcutaneous	US-1738 NCT03481816
AAV8-VRC07 (VRC-HIV AAV070-00-GT)	I	Anti-HIV-1 monoclonal antibody (VRC07)	HIV infection	Intramuscular	US-1495 NCT03374202
Ad5-CB-CFTR	I	Cystic fibrosis transmembrane conductance regulator gene	Cystic fibrosis	Intranasal	NCT00004779
LOAd703	I/II	Vector-directed cell lysis TMZ-CD40L and 4-1BBl cDNAs	Pancreatic cancer Ovarian cancer Biliary carcinoma Colorectal cancer	Intratumoral	US-1483 NCT03225989
Ad5-DNX-2401	II	Vector-directed cell lysis	Glioblastoma, Gliosarcoma	Intratumoral	US-1487 NCT03896568
Ad-p53	II	Tumor suppressor	Squamous cell carcinoma of the head and neck	Intratumoral	US-1767 NCT03544723
